# The Relationship between Seminal Fluid Hyperviscosity and Oxidative Stress: A Systematic Review

**DOI:** 10.3390/antiox10030356

**Published:** 2021-02-27

**Authors:** Federica Barbagallo, Sandro La Vignera, Rossella Cannarella, Andrea Crafa, Aldo E. Calogero, Rosita A. Condorelli

**Affiliations:** Department of Clinical and Experimental Medicine, University of Catania, 95123 Catania, Italy; federica.barbagallo11@gmail.com (F.B.); rossella.cannarella@phd.unict.it (R.C.); crafa.andrea@outlook.it (A.C.); acaloger@unict.it (A.E.C.); rosita.condorelli@unict.it (R.A.C.)

**Keywords:** seminal fluid viscosity, oxidative stress, antioxidants, male infertility, idiopathic male infertility

## Abstract

Introduction: Seminal fluid viscosity is a key parameter to achieve fertilization. Viscosity is more frequently increased in patients with infertility. However, the mechanism by which hyperviscosity causes infertility is still poorly understood. As an increased blood viscosity is associated with diseases caused by oxidative stress, it can be supposed that there is a relationship between seminal fluid viscosity and oxidative stress in male infertility. Therefore, this systematic review aims to investigate the relationship between hyperviscous seminal fluid and oxidative stress. Materials and methods: We performed a systematic search on the following databases Pubmed, MEDLINE, Cochrane, and Scopus from the earliest available date to 10 January 2021, using Medical Subjects Headings (MeSH) indexes and keywords searches. The study included all the articles that evaluated the relationship between increased seminal fluid viscosity and oxidative stress. Article reviews even though dealing with seminal fluid hyperviscosity were excluded. Results: 5 articles were included in this systematic review. The results demonstrated an important impairment of antioxidant systems and increased oxidative stress in patients with high seminal fluid viscosity. Conclusions: These findings suggest that a careful assessment of oxidative stress in patients with hyperviscosity may be very useful in clinical practice. Infertile patients with seminal fluid hyperviscosity could benefit from the treatment with antioxidants to protect sperm cells from oxidative damage and to improve their functional properties.

## 1. Introduction

### 1.1. Seminal Fluid Viscosity

The seminal rheological properties are often undervalued, although is astonishing how much information can be obtained from the physical properties of the ejaculate [1]. Viscosity is a property of a fluid that describes its resistance to flow. The ejaculate is constituted of a cell component (spermatozoa, immature germ cells, leukocytes, epithelial cells, etc.) and seminal plasma [2]. The seminal plasma is made up of fluids produced by the rete testis, epididymis, and accessory sex glands of the male genital tract [2]. The seminal plasma contains lots of components essential for the proper function of spermatozoa [2]. The first part of the semen contains the major number of spermatozoa with the fluid secreted by the epididymis [3]. The prostate secretion and then the secretions of the seminal vesicles follow subsequently [3]. Seminal fluid viscosity markedly changes in the time immediately following ejaculation [4]. After ejaculation, the semen fluid quickly coagulates into a gelatinous material to form a depository of spermatozoa in the vaginal cavity, and then it liquefies. In-vivo liquefaction occurs over a period of about 5 min but may take 20–30 min in-vitro [5,6]. Seminal vesicles secrete the seminal coagulation factors while liquefying factors derive from the prostate [7].

Seminal fluid viscosity is an important parameter for fertilization. A normal viscosity allows the migration of spermatozoa into the cervical mucus to reach the fallopian tubes [8], preserves sperm motility [9], prevents lipid peroxidation [10], and maintains sperm chromatin integrity [11].

### 1.2. Seminal Fluid Hyperviscosity and Infertility

It has been reported that seminal fluid hyperviscosity, which is characterized by a thick and coagulated appearance, occurs in 12–29% of ejaculates [12]. A retrospective study conducted by Elia et al. [13] reported that hyperviscosity occurs in 26.2% of male partners of infertile couples [13].

The seminal fluid viscosity can be estimated by aspirating the sample with a pipette and then allowing the sample to drop under the influence of gravity. The length of the threads formed is then measured. Then, according to the WHO criteria [14], the seminal fluid is classified as hyperviscous when the thread length exceeds 2 cm. However, a more rigorous method to assess seminal fluid viscosity is by the capillary tube viscometer that compares seminal fluid viscosity to that of water [1]. In their study, Elia et al. [13] classified the severity of seminal fluid hyperviscosity basing on the length of the thread. The authors reported that 13.2% of men have mild (2–4 cm), 6.6% moderate (4–6 cm), and 6.4 severe (>6 cm) hyperviscosity [13]. An increase in seminal fluid viscosity can seriously damage the components and properties of the seminal fluid [13]. It has been reported that increased viscosity occurs more frequently among infertile patients [15,16,17]. Seminal fluid hyperviscosity has been also related to a worse outcome with in-vitro fertilization and embryo transfer [18]. The mechanism by which hyperviscosity causes infertility is still poorly understood [19]. Increased semen fluid viscosity worsens sperm parameters, mainly motility [13,20]. Numerous studies have reported that higher seminal fluid viscosity is associated with asthenozoospermia due to a trapping effect of hyperviscous seminal fluid [17]. Interestingly, a decrease of chromatin integrity was also demonstrated in patients with higher semen fluid viscosity compared to controls [21].

### 1.3. Seminal Fluid Hyperviscosity and Male Accessory Gland Infection/Inflammation

Seminal fluid hyperviscosity is often associated with male accessory gland infection/inflammation (MAGI). MAGI consists of infection and inflammation of the epididymis, prostate, and/or seminal vesicles [22]. MAGI is usually the consequence of the retro-canalicular transmission of microorganisms [23]. The main etiological agents are *Neisseria gonorrhoeae*, *Chlamydia trachomatis*, and Enterobacteria [22]. MAGI rarely obstructs the seminal pathways; it mainly has a chronic paucisymptomatic course. The diagnosis of MAGI is integrated by clinical, laboratory, and ultrasound evaluation. The ultrasound examination represents a useful diagnostic tool to evaluate the site and the extension of the inflammatory process with objective criteria. Ultrasonography can also identify different forms of MAGI that impact differently on sperm parameters [23]. Indeed, infertile patients with fibrosclerotic MAGI have worse sperm parameters compared with patients hypertrophic-congestive MAGI [23]. MAGI is characterized by leukocytospermia, high levels of cytokines, and reactive oxygen species (ROS) [23]. The presence of bacteria causes the accumulation of leukocytes and an oxidative imbalance with ROS overproduction. Then, the generation of pro-inflammatory cytokines modulates the activity of pro- and anti-oxidative systems [22]. Interestingly, the end products of oxidative stress may persist in the semen also after the eradication of infection, further impairing the function of spermatozoa [22].

A strong association between MAGI and infertility has been reported [24]. The alterations of sperm parameters are directly associated to the anatomical extension of MAGI [25]. Indeed, the sperm quality of patients with MAGI involving the prostate, seminal vesicles, and epididymis is worse compared with patients who have only prostatitis [25]. Furthermore, the bilateral involvement of the sex glands is associated with worse sperm parameters [25]. Previous studies reported that the seminal fluid viscosity of patients with MAGI was significantly higher than that in the controls [26]. Interestingly, the increase of viscosity has been correlated with the anatomic extension of MAGI. Indeed, patients with prostate-vesicular-epididymitis, show seminal fluid viscosity significantly higher compared to patients with prostatitis alone and prostate-vesiculitis [26]. On the contrary, another study did not find any association between seminal fluid viscosity and the presence of microorganisms in semen cultures [27]. Therefore, seminal fluid hyperviscosity does not necessarily conceal a genitourinary tract infection [27].

Leukocytes can also have an important role in the development of hyperviscosity [12]. Patients with higher seminal fluid viscosity have increased concentrations of leukocytes compared to patients with normal viscous semen. Mahran and colleagues reported an increase of leukocytes in 37.5% of infertile men with high seminal fluid viscosity [28]. Moreover, leukocytospermia correlates with a reduction of sperm motility and vitality in patients with hyperviscous seminal fluid [28].

### 1.4. Seminal Fluid Hyperviscosity and Anti-Sperm Antibodies

Increased viscosity is also associated with the presence of anti-sperm antibodies [29]. The existence of anti-sperm antibodies was first reported by Rumke and Wilson in 1954 [30,31] and since that time anti-sperm antibodies have been studied as a possible cause of infertility. The presence of anti-sperm antibodies was found in up to 9–12% of infertile couples [32]. However, these antibodies are present in approximately 1–2.5% fertile men [33,34] and 4% fertile women [35]. This indicates that the presence of anti-sperm antibodies alone does not impair the fertilization process and that not all anti-sperm antibodies cause infertility [36].

The anti-sperm antibodies are formed against antigenic proteins following the exposure of these proteins to immune cells [37]. The testes are an immunologically privileged site because of the presence of the blood–testis barrier that protects the autoantigenic germ cells from immune attacks [38]. However, several factors, including environmental toxicants or genetic disorders can impair the integrity of the blood-testis barrier [38]. In men, the risk of the development of anti-sperm antibodies increases after blood-testis barrier breakdown, surgical trauma, infections, testicular tumor, and varicocele [37]. MAGI can cause the formation of anti-sperm antibodies yielding an autoimmunity response [39,40]. Anti-sperm antibodies can interfere directly or indirectly with various steps of human fertilization, such as acrosome reaction, capacitation, fertilization, and implantation [36].

The process of anti-sperm antibody generation is poorly understood and its role in fertility is still controversial. Numerous studies indicate that couples with anti-sperm antibodies have lower pregnancy rates than couples without anti-sperm antibodies [36]. Moreover, an inverse relationship between the titer of spermatozoa bound to anti-sperm antibodies and the fertilizing capability of sperm has been shown [36]. However, some researchers reported that there was no association between anti-sperm antibodies and the pregnancy rate [37]. The unclear role of anti-sperm antibodies in human fertility could reflect the inadequacies of the diagnostic techniques that detect antibody binding to spermatozoa but do not evaluate their antigenic specificities [36].

Interestingly, an increased seminal fluid viscosity has been associated with the presence of sperm antibodies [29]. Moulik and colleagues studied 96 patients who underwent assisted reproductive technique programs. They evaluated the presence of sperm antibodies in seminal plasma by ELISA technique and the relation between anti-sperm antibodies and the seminal fluid viscosity. The results of this study showed the presence of anti-sperm antibody IgA and/or IgG in 19 of them and 15 of this latter (79%) had also abnormally high seminal fluid viscosity. The prevalence of anti-sperm antibodies in samples with high viscosity was significantly greater than in samples with normal viscosity [29].

### 1.5. Seminal Fluid Hyperviscosity and Oxidative Stres

Interestingly, some studies have reported a correlation between hyperviscosity and oxidative stress [12]. Seminal fluid hyperviscosity leads also to low levels of seminal plasma fructose, ascorbic acid, calcium, and zinc. Fructose and ascorbic acid are mainly produced by the seminal vesicles, whereas calcium and zinc are biomarkers of prostatic activity [12]. These trace elements are important for spermatogenesis, energy production, defence against ROS, and the fertilization process [12]. Zinc plays an important role as a cofactor for several antioxidants such as Cu/Zu-superoxidative dismutase. Thus, zinc deficiency in patients with seminal fluid hyperviscosity can contribute to oxidative stress and, in turn, to sperm quality [12].

### 1.6. Oxidative Stress and Male Fertility

Infertility is defined as the failure to achieve a clinical pregnancy after 12 months or more of unprotected sexual intercourse (WHO, 1983) [41]. Infertility affects 15% of all couples in the reproductive age in industrialized countries [42]. A male factor is responsible for about half of the cases of infertility [42]. However, the etiology of male infertility is unexplained in up to 75% of the patients [43]. This condition is defined as “idiopathic infertility” [44].

In the last decades, a large body of literature has shown that oxidative stress is one of the main causes of male infertility [45]. A significant number of cases of male infertility, classified as “idiopathic”, may instead be due to an increased level of oxidative stress [46]. In fact, it is well known that infertile patients have higher reactive oxygen species (ROS) levels in their seminal fluid compared with fertile men [47]. ROS are products of normal cellular metabolism. These include the superoxide anion radical, the hydroxyl radical, the peroxyl radical, and free radicals derived from nitrogen (such as nitric oxide, peroxynitrite, nitroxyl anion, and peroxynitrous acid) [48]. The superoxide anion radical seems to be the principal ROS produced by spermatozoa [48]. ROS plays an important role in sperm capacitation and acrosome reaction at physiological levels. In contrast they can oxidize, and in turn, damage DNA, proteins, and lipids at high concentrations [49]. The human seminal fluid contains numerous systems with antioxidant activity to contrast the negative effects of ROS including the superoxide dismutase (SOD), the glutathione peroxidase/reductase system (GPX), and catalase (CAT). The antioxidant system is also composed of non-enzymatic antioxidants such as vitamins C and E, Zn, Cu, and glutathione [46]. However, an imbalance between oxidant production and antioxidants capacity increases oxidative stress. An increase in oxidative stress plays a main role in a variety of diseases, including infertility. Oxidative stress can also alter the seminal plasma proteome [50]. Thus, specific proteins can be overexpressed in patients with diseases characterized by increased levels of oxidative stress in the seminal fluid [50]. Accordingly, it is noteworthy that some seminal plasma proteins have been suggested as possible markers of the damage caused by oxidative stress in infertile patients [50].

Spermatozoa are particularly sensitive to the detrimental effects of increased oxidative stress because their plasma membrane is constituted by high levels of polyunsaturated fatty acids (PUFA). The double bonds of the membrane lipids can be oxidized by ROS and this causes a process called “lipid peroxidation” and in turn, a reduction of membrane fluidity. This impairs sperm motility [51] and its ability to fertilize the oocytes. One of the end-products of lipid peroxidation is malondialdehyde (MDA) [52]. MDA has been used in biochemical assays to evaluate the severity of peroxidative damage in spermatozoa [45]. Moreover, ROS can directly damage sperm DNA [52]. Indeed, spermatozoa are unable to repair DNA due to the lack of the cytoplasmic enzyme systems involved in DNA repair molecular mechanisms [52]. On this basis, antioxidant therapies have been developed for the treatment of male infertility due to increased oxidative stress. A large number of molecules with antioxidant properties have been developed and are commonly used in clinical practice. However, the latest Cochrane systematic review reported that many studies published are of poor quality [53]. According to a position statement by the Italian Society of Andrology and Sexual Medicine, the therapeutic value of antioxidants increases in patients with idiopathic infertility characterized by alteration of sperm parameters and sperm DNA fragmentation after a proper diagnostic workup [54].

### 1.7. Aim of the Study

Seminal fluid viscosity is an important parameter for fertilization. Previous studies reported that an increased seminal fluid viscosity occurs more frequently among infertile male [15,16,17]. However, the mechanism by which seminal fluid hyperviscosity causes male infertility is still poorly understood. Several causes have been proposed, including MAGI [25], the presence of anti-sperm antibodies [29], and leukocytospermia [12].

It is widely demonstrated that oxidative stress has a main role in the pathogenesis of male infertility [45]. Interestingly, oxidative stress has been also associated with blood hyperviscosity [55,56,57]. Increased levels of MDA and protein carbonyl have been observed in patients with higher blood viscosity [55]. MDA, at different concentrations, can increase significantly the viscosity of erythrocytes [58]. In fact, it has been shown that MDA can cross-bind or induce secondary oxidative damage in the plasma proteins. Small aldehydes can react with membrane proteins and modify their structure [59]. Higher blood viscosity has also been associated with systemic diseases associated with oxidative damage, such as diabetes mellitus, coronary artery disease, hyperlipoproteinemia, and chronic kidney disease [60,61].

Thus, as increased blood viscosity is associated with diseases caused by increased oxidative stress, an association between seminal fluid hyperviscosity and seminal oxidative damage can be hypothesized in infertile patients. On this basis, we conducted this systematic review to investigate the relationship between seminal fluid hyperviscosity and oxidative stress.

## 2. Materials and Methods

### 2.1. Sources

Databases were independently searched by F.B and R.A.C. We performed a systematic search through Pubmed, MEDLINE, Cochrane, and Scopus databases from the earliest available date to 10 January 2021, using Medical Subjects Headings (MeSH) indexes and keywords searches. The search strategy included the following combination of Medical Subjects Headings (MeSH) terms and keywords: “seminal viscosity”, “semen viscosity”, “sperm viscosity”, “oxidative stress”, “hyperviscosity”, “sperm lipid peroxidation”, “8-hydroxydeoxyguaosine”, “antioxidants”. Additional manual searches were made using the reference lists of relevant studies. Only articles in English reporting complete data of clinical relevance for the present review and published up to 10 January 2021 were included in the analysis. All abstracts and relevant full texts were evaluated. Two authors independently (F.B. and R.A.C.) reviewed the abstracts and selected only the articles that were pertinent to the objective of this study. Any disagreement was resolved by discussion with the other investigators. The reference lists of the identified articles were also used to find pertinent studies.

### 2.2. Study Selection

The study included all the articles that evaluated the relationship between increased seminal fluid viscosity and oxidative stress. Moreover, all the studies investigating the effects of antioxidant therapy simultaneously on seminal viscosity and sperm oxidative stress were also included. Article reviews even though dealing with seminal fluid hyperviscosity were excluded. Review articles were used only as a source for the discussion. Duplicates were carefully checked and removed.

## 3. Results and Discussion

**Figure 1:**
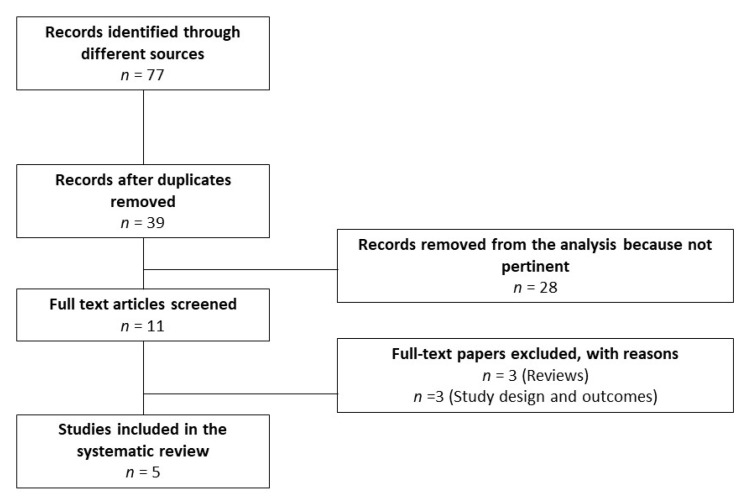
Flowchart of the studies included in systematic review.

The aforementioned search strategy identified a total of 77 records. After the exclusion of 38 duplicates, the remaining 39 articles were screened. Of these, 28 were judged not pertinent. Among the remaining studies, 3 were excluded because they were reviews and 3 were excluded for the study design and outcomes. Finally, 5 articles were included in this study [8,19,62,63,64] (Figure 1).

These articles were then classified into those investigating the relationship between increased seminal fluid viscosity and oxidative stress [8,19,62,63] and only one was found investigating the effects of the antioxidant therapy simultaneously on seminal viscosity and sperm oxidative stress [64]. We tabulated the articles considering the number of the patients, the type and the aim of the study, the methods of assessment of both viscosity and oxidative stress, and the main findings (Table 1).

### 3.1. Seminal Fluid Hyperviscosity and Oxidative Stress

In 2001, Siciliano and colleagues demonstrated, for the first time, an important impairment of antioxidant systems in hyperviscous ejaculates [62]. They assessed seminal fluid antioxidant capacity of 120 patients subdivided into four groups: asthenozoospermia (*n* = 40), oligoasthenozoospermia (*n* = 50), hyperviscosity and asthenozoospermia (*n* = 14), hyperviscosity and oligoasthenozoospermia (*n* = 16). A group of 25 healthy donors with normozoospermia was used as control. They evaluated seminal superoxide dismutase activity and catalase activity to assess the scavenger antioxidant capacity against ROS, whereas the chain-breaking antioxidant activity was evaluated by measuring the total antioxidant capacity (TAC). Interestingly, they found that asthenozoospermic and oligoasthenozoospermic patients with seminal fluid hyperviscosity had lower activity of both the scavenger and chain-breaking antioxidant systems [62]. Catalase and total antioxidant capacity values were also decreased in patients with hyperviscous ejaculates compared with controls. They also evaluated seminal zinc and fructose concentrations to exclude prostatic or vesicular hypofunction, and the presence of polymorphonuclear (PMN) granulocytes but no significant differences were found among the four groups [62].

In 2008, Adeymir and colleagues measured the concentrations of malondialdehyde (MDA) and protein carbonyls in the seminal plasma and spermatozoa of 60 infertile patients and 42 controls [63]. Infertile patients enrolled in this study had at least one sperm parameter altered, semen leukocyte count was within the normal values (<1 × 10^6^/mL), and no anti-sperm antibody. Correlation analysis revealed a significant positive relationship between seminal fluid viscosity with seminal plasma or sperm MDA (r = 0.676, *p* < 0.01; r = 0.482, *p* < 0.01, respectively) in infertile patients. Besides, seminal plasma viscosity correlated with sperm and seminal plasma protein carbonyl concentrations (r = 0.276, *p* < 0.05; r = 0.308, *p* < 0.05, respectively). In contrast, no significant correlation was found in the control groups. They hypothesized that the cause of the increased viscosity in these patients was a modification of the interactions between proteins [63]. In fact, lipoperoxidation-derived aldehydes, such as MDA, can bind to proteins, modifying them, and generating covalent adducts; in turn, these structures can undergo further spontaneous modifications [59]. In contrast with these findings, Layali and colleagues reported higher levels of MDA in the seminal plasma of fertile men compared to infertile patients with or without hyperviscosity [8]. On the other hand, accordingly with other studies, they found that infertile patients with hyperviscous semen had significantly lower TAC levels compared with infertile patients without seminal fluid hyperviscosity or controls [8].

Interestingly, in both the studies by Siciliano et al. and Aydemir et al. leukocytes levels were within the normal range. It is known that leukocytes and abnormal spermatozoa are the main endogenous sources of oxidative stress in the seminal fluid [12]. Moreover, seminal fluid hyperviscosity has been correlated to infections or inflammation of the genital tract. We have reported that patients with male accessory gland infection had a higher viscosity compared to normal controls [26]. Moreover, the severity of the hyperviscosity correlated with the extension of the inflammatory process of the male accessory glands [26]. Despite being an aspect poorly investigated, previous studies have also evaluated the relationship between seminal viscosity and the concentrations of pro-inflammatory cytokines in the seminal fluid. The results of these studies have shown that sperm hyperviscosity correlated positively with the oxidative stress and pro-inflammatory interleukins, TNF-α (r = 0.76; *p* < 0.01) or IL-6 (r = 0.561; *p* < 0.05) in patients with male accessory gland infections [19]. Moreover, they reported that the use of viscometer allowed to obtain a quantitative evaluation of seminal fluid hyperviscosity compared to the methods suggested by the criteria of the World Health Organization (WHO) manual for the semen analysis [14]. Thus, they showed that the measurement of seminal fluid viscosity using a viscometer allowed to estimate the severity of hyperviscosity and, consequently, it may give an idea of the extension of MAGI in the male accessory glands [19].

In the last years, the role of antioxidants in the therapy of male infertility has has been widely debated. Several molecules are commonly used in clinical practice in an attempt to improve sperm parameters. Among antioxidants, N-acetylcysteine (NAC) has been reported to improve both seminal fluid viscosity and to decrease sperm oxidative stress [64]. A randomized clinical trial evaluated the effect of NAC on sperm parameters and the antioxidant system in 120 patients with idiopathic male infertility [64]. This study showed that patients who received NAC (600 mg/day, orally) had a significant improvement in both seminal fluid viscosity, serum TAC, and total peroxide and oxidative stress index compared to controls. Patients treated with NAC had also a significant improvement of sperm motility that could be attributable to the decreased ROS production and seminal fluid viscosity. Previous studies have reported that NAC, acting as a scavenger of hydroxyl radicals, significantly decrease ROS production in human sperm samples [65]. Moreover, NAC lowers the viscosity of the cervical mucus by acting on the disulfide bonds [66]. Unexpectedly, our systematic research did not find other studies which have specifically evaluated the effects of antioxidant treatment simultaneously on viscosity and oxidative stress. Further studies into the effects of antioxidants treatment on seminal viscosity could be very useful to improve the therapeutic strategy of male infertility.

### 3.2. Assessment of Seminal Fluid Viscosity

As discussed in Section 1.1, seminal fluid viscosity is usually estimated by aspirating the sample into a pipette and observing the length of the threads formed while the sample drops by gravity [14]. However, a capillary tube viscometer can be also used to evaluate seminal fluid viscosity [1]. Table 1 reports the methods of evaluation of seminal fluid viscosity used in the studies included in this review Interestingly, when using the WHO standard method for the assessment of seminal fluid viscoelasticity, all the patients Aydemir and colleagues studied had normoviscous results. However, viscosity measured using a capillary viscometer showed a statistically significant difference between the group of patients with infertility who had hyperviscosity and that of the controls who had normal viscoelasticity. Thus, the authors suggested that the qualitative evaluation through glass pipettes might be inefficient to estimate the differences in seminal fluid viscosity [63].

## 4. Limitations and Future Perspectives

Based on these studies, the relationship between seminal viscosity and oxidative stress is quite clear. However, some limitations must be taken into account. Firstly, few studies have examined the relationship between seminal fluid viscosity and sperm oxidative stress. Indeed, only 5 studies were obtained after a systematic search was carefully conducted. It is not possible to establish a cause-effect relationship between seminal fluid hyperviscosity and oxidative stress since almost all of them are observational studies. Moreover, the studies result to be heterogeneous even for the methods of assessment of both viscosity and oxidative stress (Table 1). We found only one study which has specifically evaluated the effects of antioxidant treatment simultaneously on viscosity and oxidative stress. Certainly, further studies exploring the biochemical mechanisms by which oxidative stress leads to seminal fluid hyperviscosity and the effects of antioxidants treatment on seminal viscosity will be very useful to better understand this relationship and to improve the therapeutic strategy of male infertility.

## 5. Conclusions

**Figure 2:**
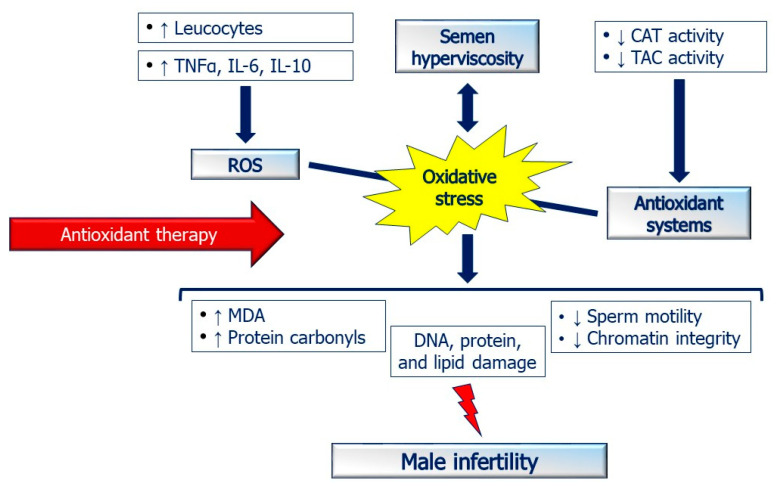
Seminal fluid hyperviscosity and oxidative stress. The mechanism by which hyperviscosity causes infertility is still poorly understood. Increased seminal fluid viscosity worsens sperm parameters, especially motility, and decrease chromatin integrity [35]. Interestingly, previous studies have shown an imbalance between oxidant production and antioxidant capacity in patients with high seminal fluid viscosity. A decreased activity of catalase (CAT) and total antioxidant capacity (TAC) have been reported in patients with hyperviscous ejaculates [8,62]. On the contrary, seminal fluid hyperviscosity correlated to increased levels of malondialdehyde (MDA) and protein carbonyls [63], high levels of pro-inflammatory interleukins, and leukocytes [19]. The increased oxidative stress damages DNA, proteins, and lipids and in turn, impairs male fertility. Thus, a careful assessment of oxidative stress in patients with hyperviscosity may be very useful in clinical practice. Infertile patients with seminal fluid hyperviscosity could benefit from the treatment with antioxidants to protect sperm cells from oxidative damage and to improve their functional properties.

Seminal fluid hyperviscosity can be an important cause of poor sperm quality. Previous studies have shown increased oxidative stress in patients with seminal fluid hyperviscosity (Figure 2). In clinical practice, several conditions are associated with seminal fluid hyperviscosity that may alter sperm parameters, including idiopathic infertility [67], varicocele in particular when associated with dilation of the periprostatic venous plexus [68], and obesity [69]. Moreover, an increased seminal fluid viscosity can persist also after bacterial eradication in patients with previous urogenital infections [22]. Therefore, it is always necessary to evaluate the rheological properties of patients even after bacterial eradication because of the risk of maintaining an isolated alteration of seminal fluid viscosity [22].

The exact mechanisms by which seminal fluid hyperviscosity is associated with oxidative stress are still not clear. Seminal fluid hyperviscosity may increase oxidative stress or it can reflect a condition or a disease that provokes an imbalance between oxidative and antioxidant systems. However, although we do not know “which came first: the chicken or the egg”, seminal fluid hyperviscosity should alert the clinician to check for oxidative damage. In clinical practice, these findings suggest that a careful assessment of the oxidative stress in patients with seminal hyperviscosity may be very useful. Indeed, infertile patients with seminal fluid hyperviscosity could benefit from the treatment with antioxidants to protect sperm cells from the damage resulting from increased oxidative stress and to improve their functional properties.

## Figures and Tables

**Table 1:** Studies evaluating the relationship between seminal fluid viscosity and sperm oxidative stress.

Authors/Year/Title	Type of Study	Patients	Aim of the Study	Assessment of Seminal Fluid Viscosity	Assessment of Sperm Oxidative Stress	Main Findings
Siciliano et al., 2001 *Impaired seminal antioxidant capacity in human semen with hyperviscosity or oligoasthenozoospermia* [62]	Cross sectional study	120 infertile patients subdivided in four groups: - AA (*n* = 40) - OA (*n* = 50) - VA (*n* = 14) - VOA (*n* = 16) 25 healthy controls	To investigate the seminal enzymatic and non-enzymatic antioxidant capacity in with A, O, hyperviscosity or a combination of these	WHO 1999	CAT activity, TAC and SOD activity	CAT activity and TAC values were significantly decreased in patients with hyperviscous ejaculates compared with controls.
Aydemir et al., 2008 *The influence of oxidative damage on viscosity of seminal fluid in infertile men* [63]	Observational study	60 infertile patients 42 healthy controls	To investigate whether oxidative damage was associated with seminal plasma viscosity in infertile patients	WHO 1999, and by Viscometer	Levels of MDA and protein carbonyls in sperm and seminal plasma	Seminal plasma viscosity significantly correlated with sperm and seminal plasma MDA and protein carbonyl concentrations in infertile patients.
Ciftci et al., 2009 *Effects of N-acetylcysteine on semen parameters and oxidative/antioxidant status* [64]	RCT	120 patients with idiopathic male infertility, randomly in two groups: - The study group (*n* = 60) received NAC, 600 mg/day, orally for 3 months - The control group (*n* = 60) received a placebo	To examine whether NAC has a beneficial effect on semen parameters and oxidative status in idiopathic male infertility	WHO 1999	TAC, TP level and OS index	Patients who received NAC had a significant reduction in both seminal fluid viscosity, TP level and OS index, whereas the TAC significantly improved.
Castiglione et al., 2013 *Relationship of semen hyperviscosity with IL-6, TNF-α, IL-10 and ROS production in seminal plasma of infertile patients with prostatitis and prostato-vesiculitis* [19]	Observational study	169 infertile patients - With chronic bacterial prostatitis (*n* = 74) - With bilateral prostato-vesciculitis (*n* = 95) 42 healthy fertile men	To analyze whether seminal fluid viscosity is associated with ROS, levels of cytokines (TNF-alpha), IL-6 and IL-10 and seminal leucocyte concentration, and whether ROS production is related to the extent of inflammation	Viscometer	ROS production, levels of cytokines (TNFα, IL-6, and IL-10), and seminal leucocyte concentration	Sperm hyperviscosity correlated positively with the oxidative stress and pro-inflammatory interleukins, TNF-α and IL-6 in patients with male accessory gland infections.
Layali et al., 2015 *Total antioxidant capacity and lipid peroxidation in semen of patient with hyperviscosity* [8]	Cross sectional study	25 infertile patients with normal viscosity 22 infertile patients with hyperviscosity 12 healthy fertile men	To evaluate seminal plasma total antioxidant capacity and malondialdehyde levels in infertile patients with hyperviscoys and non hyperviscous semen samples	WHO criteria (unspecified edition; assumed 2010)	TAC and MDA levels	Infertile patients with hyperviscous semen had significantly lower TAC levels compared with infertile patients without semen hyperviscosity or controls.

Abbreviations: AA = asthenozoospermia; OA = oligoasthenozoospermia; VA = hyperviscous asthenozoospermia; VOA = hyperviscous oligoasthenozoospermia; MDA = malondialdehyde; SOD = superoxide dismutase; NAC = N-acetylcysteine, OS = oxidative stress; ROS = reactive oxygen species; TAC = total antioxidant capacity, TP = total peroxide, RCT = randomized controlled trial.
